# Targeted mentoring for human immunodeficiency virus programme support in South Africa

**DOI:** 10.4102/sajhivmed.v20i1.873

**Published:** 2019-02-14

**Authors:** Geoffrey Jobson, Moyahabo Mabitsi, Jean Railton, Cornelis J. Grobbelaar, James A. McIntyre, Helen E. Struthers, Remco P.H. Peters

**Affiliations:** 1 Anova Health Institute, South Africa; 2 School of Public Health and Family Medicine, University of Cape Town, South Africa; 3 Division of Infectious Diseases and HIV Medicine, Department of Medicine, University of Cape Town, South Africa

## Abstract

**Background:**

Mentoring is a required component of health systems strengthening technical assistance interventions in low- and middle-income countries (LMICs). Mentoring is useful because it does not necessarily compromise service delivery and promotes the sharing of newly acquired knowledge and skills. However, there is a lack of research on the implementation of mentoring in the context of the HIV epidemic in southern Africa.

**Objectives:**

This qualitative evaluation focussed on understanding the implementation process of targeted mentoring for clinical practice, data management and pharmacy management, at public health care facilities in South Africa; and on identifying critical factors influencing the effectiveness of mentoring as a technical assistance intervention in this context.

**Methods:**

Purposive sampling was used to select participants from public health facilities in three South African Provinces. Participants were invited to take part in structured interviews. Datawere analysed using thematic analysis, and two core themes were identified: mentoring as knowledge and skills transfer; and mentoring as psychosocial support.

**Results:**

In terms of knowledge and skills transfer, the sequential implementation of proactive and reactive mentoring was critical. Initial proactive mentoring involved mentors initiating training and developing professional relationships with mentees. Thereafter, a reactive mentoring phase allowed mentees to request support when required. This enabled mentors to leverage real-world problems faced by health workers to support their implementation of new knowledge and skills. The availability and accessibility of mentors alongside the relationships between mentors and mentees provided psychosocial support for health care workers which facilitated their self-efficacy in implementing new knowledge and skills.

**Conclusion:**

These findings suggest that the success of mentoring programmes in LMICs may require specific attention to both knowledge transfer and the management of interpersonal relationships.

## Introduction

The mentoring of clinical staff has become an important aspect of the human immunodeficiency virus (HIV) response in many low- and middle-income countries (LMICs). Using mentors to support local staff enables implementing agencies and governments to target specific cadres of health workers to develop and entrench the skills and knowledge required to successfully implement interventions.^1^ Mentoring has also played an important role in supporting task shifting of particular functions from doctors to mid-level staff such as nurses, counsellors and medical officers.^1^ Using mentoring as a component of technical assistance interventions is beneficial because it does not compromise service delivery and promotes the sharing of newly acquired knowledge and skills.^2^ Ndwiga et al. also note that workplace mentorship has been found to increase confidence and self-esteem, decrease stress and conflict, and improve job satisfaction among mentees.^2^

While mentoring has the potential to significantly improve service provision, there are a number of important features of the mentor-mentee relationship that may affect the relative success of such programmes. Straus et al. for example, found that successful mentoring relationships were characterised by reciprocity, mutual respect, clear expectations, a personal connection and shared values.^3^

Factors related to the context of local health systems may also affect the success or failure of mentoring initiatives. In Kenya, for example, Ndwiga et al. found that the effectiveness of mentoring was affected by erratic supplies of medication and commodities, high client caseloads and staff shortages.^2^

Mentoring programmes supporting the scale-up of HIV treatment in sub-Saharan Africa (SSA) have frequently focussed either on mentoring clinical staff or on supporting administrative and logistical processes.^1,4,5,6^ While these programmes have shown impressive results, their focus on either clinical or administrative processes may lead to constraints in their effectiveness. Therefore, we explore the role of targeted mentoring using a needs-based approach to support both clinical and some administrative processes as part of a technical assistance package targeting HIV and tuberculosis (TB) services at public healthcare facilities in three provinces of South Africa.

## Methods

### Study setting

The study was conducted at selected health facilities in Limpopo, Gauteng and the Western Cape. Facilities in Limpopo and the Western Cape were situated in rural districts, while the Gauteng facilities were located in peri-urban townships. All facilities had been receiving technical assistance from Anova Health Institute (hereafter referred to as ‘the implementing agency’) since 2010, and most had previously been sites where the agency had directly provided HIV-related services.

The implementing agency’s HIV and TB-related health systems strengthening (HSS) support activities were primarily funded by USAID/PEPFAR. In 2009, PEPFAR’s focus shifted away from direct service provision towards technical assistance and HSS.^7^ Hence, the focus of the implementing agency’s work between 2009 and 2014 was on HSS and the provision of technical support to the South African Government’s HIV response, with staff mentoring forming a central aspect of this support. Mentoring was provided to nurses to assist with the implementation of nurse initiated and managed ART (NIMART); to data capturers and administrators to assist with managing the ART (antiretroviral therapy) programmes reporting requirements; and to pharmacy staff to support efficient stock control and pharmacy management.

### Theoretical framework

This study was informed by literature focussing on mentoring as a broad approach to individual and organisational development in various contexts,^8,9^ as well as research focussing more specifically on mentoring in the context of technical assistance programmes in LMICs.^2,5,10^ This literature was reviewed in reference to the World Health Organization’s 2006^11^ recommendations on the use of clinical mentoring to support HIV programme implementation in resource-limited settings.

### Study design

This study is a qualitative evaluation of the role of mentoring as part of a technical assistance package targeting HIV and TB services as provided by the implementing agency at public healthcare facilities in three South African health districts.

Structured interviews to explore health workers’ perceptions of the implementing agency’s mentoring programme were conducted by independent consultants with 74 participants (both male and female) from government health facilities in Limpopo Province, Gauteng and the Western Cape. Interviews were conducted in person, in private rooms at individual facilities, between October 2014 and July 2015. Refusal rates were very low, with only five participants refusing to participate in interviews as a result of repeatedly being unavailable or having to attend to urgent medical problems in their facilities. Participants were purposively selected based on their involvement in the technical assistance programme. Interviews were conducted with facility managers and their deputies, nurses, pharmacists and data staff. While the evaluation included questions relating to the entire technical assistance package, this article focusses on understanding healthcare workers’ perceptions of the mentoring component of the package.

Signed informed consent was obtained from all participants prior to their participation. To maintain anonymity of respondents, no identifying information was collected. The study protocol and materials were approved by the University of the Witwatersrand’s Human Research Ethics Committee (M120625).

### Data analysis

Interviews were audio-recorded and transcribed verbatim. Data were managed using NVIVO 11^12^ and were analysed using a thematic analysis approach.^13^ Firstly, interview transcripts were read carefully while paying particular attention to discussion related to the mentoring programme. Once the two authors responsible for data analysis were familiar with the data set, initial coding was conducted independently by each author to identify units of text with relevance to the research topic. Text units dealing with the same issue were grouped together in an initial set of themes and 48 basic themes were interpreted from the data in this stage. The same unit of text could be included in more than one theme.

These themes were then discussed and refined in relation to both the coded content of the basic themes, and the data set as a whole, in order to create a coherent set of organising themes. Five organising themes were produced in this stage of analysis. Finally, these organising themes were analysed to develop two main themes related to the role of the implementing agency’s mentoring programme in implementing HIV-related services. These were: (1) targeted mentoring as knowledge and skills transfer and (2) mentoring as psychosocial support.

## Ethical consideration

Signed informed consent was obtained from all participants. The study protocol was approved by the University of the Witwatersrand’s Human Research Ethics Committee (reference number: M120625).

## Results

### Mentoring as knowledge and skills transfer

The targeted mentoring programme focussed on supporting three main aspects of the South African Department of Health’s (DoH) antiretroviral therapy (ART) scale up. These were: NIMART, pharmacy management and data management. The mentoring programme adopted a two-stage approach, which we classified as proactive and reactive mentoring.^14^

### Proactive mentoring

Proactive mentoring involved scheduled visits to health facilities by mentors. During these visits, several activities took place, which were noted as important by the DoH staff. For professional nurses being trained to initiate ART, an important feature of the mentoring visits was having nurse-mentors accompany them during consultations with patients. In this role, mentors were able to provide bridging support between off-site training courses and the actual implementation of the skills learnt on these courses. This was particularly important because initiating and managing ART was a significant increase in nurses’ responsibilities in the context of HIV treatment and care:

‘I think the mentoring system is good and it is needed … ART initiation is difficult so the mentoring has helped.’ (Nurse 1, Johannesburg)

Supporting nurses in learning to initiate ART, and to manage the care of patients on ART, was a critical feature of participants’ discussions about the role of the mentoring programme, and several participants discussed the knowledge they had gained through the training and mentoring process as empowering them to take on a more active role in treating HIV-positive patients required by the NIMART programme:

‘… before I was scared of patients who had HIV/AIDS, I was scared and immediately after I was trained I had power and now I can do it and even critically ill patients are living healthy lives.’ (Deputy facility manager 1, Limpopo)

Several participants also discussed support from the implementing agency in terms of their mentors’ roles in assessing and evaluating their practice. In this context, the mentors’ presence facilitated the application of the skills learnt through NIMART training:

‘And [*mentors*] come for mentoring us for treatment and checking if we have difficulties. If I have a patient and want to initiate and I am taking the baseline to find the patient is in bad condition they check if we are coping.’ (Nurse 3, Limpopo)

The mentors’ roles in assessing participants’ progress was also discussed by pharmacists, pharmacy assistants and data staff as an important aspect of the mentoring programme. In assessing the application of knowledge and skills, the mentors created a platform from which to provide ongoing training and support to these staff. This process of ‘checking’ was an important means of jointly identifying areas in which staff needed more support:

‘… mostly [*mentors*] come to check how we pack treatment and make sure that if over-stocking arrange the drugs so we use the old ones first, and they come and check the data capturer …’ (Deputy facility manager 2, Johannesburg)

‘They do the stock checks of ARVs and the other stock. They also available on the phone to help with stock control. [*Name*] helps to ensure the stock in the pharmacy is arranged logically.’ (Pharmacist 1, Winelands)

‘We get support from [*the implementing agency*]. When they come and check the computers and check the data and check the data is clear.’ (Data capturer 1, Johannesburg)

While proactive mentoring was generally appreciated by the DoH staff, for the mentoring programme to operate successfully in this role, clear interpersonal communication and relationship management on the part of the mentors were critical. Where these factors were perceived to be absent, the DoH staff found the programme less useful:

‘You just see them here and they say they here for support visit but there is no programme around their visits as we don’t know when they are coming and cannot plan around it. They just come and you might not be ready for them and therefore the visit does not become fruitful. If we knew when they were coming then we could plan the problems we are having.’ (Facility manager 1, Limpopo)

‘They don’t consult with me and that is one of the challenges.’ (Facility manager 1, Johannesburg)

‘They have not told us that they are not capturing the data anymore.’ (Data capturer 2, Johannesburg)

A further challenge in terms of proactive mentoring occurred when implementing agency staff took on a direct service delivery role, and in doing so prevented the DoH staff from applying their training. A participant in the Winelands, for example, reported that the nurse mentor tended to do the clinical work herself, rather than allowing her mentees to practice and gain experience:

‘[*Name*] does the work but does not explain what she is doing … she does more clinical work than a mentor should do.’ (Facility manager 3, Winelands)

### Reactive mentoring

A second theme highlighted from study participants’ discussion around the mentoring programme related to mentors’ roles as problem solvers and sources of on-demand support. We termed this reactive mentoring.^14^ To facilitate this, mentors provided mentees with their contact information to enable them to ask for assistance as and when they needed it. This aspect of the mentoring programme was important as an enabling mechanism for participants, particularly for nurses who were beginning to initiate and manage patients on ART:

‘… we phone if we have problems and when they come there are issues we ask them and they come frequently and help us with problems and how do we deal with it.’ (Facility manager 4, Johannesburg)

‘[*The mentor*] gives technical support whenever I have a problem, I call or they visit and whatever challenges I face they come.’ (Data capturer 2, Limpopo)

In this reactive role, mentors were able to facilitate skill building and knowledge transfer by ensuring that they were available to assist the DoH staff when the need arose; and participants noted that mentors used the opportunities provided by the DoH staff contacting them to build on the knowledge they gained through training:

‘Last week there was a patient here and we called [*Name*] and she consulted with another doctor and came back to me to do in-service training and teach us about the HIV virus and how deal with the patients.’ (Facility manager 1, Limpopo)

‘[*Name*] is my mentor and she regularly comes to check my defaulter list – every problem I come across she is there to help … there was a duplication of patients and she helped.’ (Data capturer 3, Gauteng)

While the DoH staff clearly valued the role of the implementing agency’s mentors, the implementation of reactive mentoring required careful attention to the level and type of assistance requested by healthcare workers to ensure that mentees continued to develop their own skills and did not become overly dependent on their mentors, for example:

‘They help me with a lot of things I cannot work without them.’ (Nurse 4, Johannesburg)

A second challenge in terms of reactive mentoring was that because they were each responsible for several health facilities, mentors were sometimes unavailable when their help was needed. This had the potential to become problematic as it could result in a breakdown in trust between mentors and mentees:

‘They used to come but now that they have to service other clinics it is minimal.’ (Nurse 7, Limpopo)

Both proactive and reactive mentoring contributed to the ability of the DoH staff to implement the knowledge and skills gained through attending training courses and workshops. However, in addition to this, our interpretation of participant interviews suggested that the mentorship programme also played an important empowering role for staff by providing a source of psychosocial support as they took on new responsibilities.

### Mentoring and psychosocial support

The role of mentors in providing psychosocial support was most important among nurses who were beginning to initiate ART. There were several ways in which the mentorship programme provided psychosocial support to these staff. Firstly, simply knowing that mentors were available to assist in problem-solving was important in enabling participants to implement their training. The idea that their mentors were ‘just a phone call away’ allowed them to take on this role without undue fear that they would cause harm to their patients:

‘With complicated cases I like to know that [*I*] can phone [*the implementing agency*].’(Facility manager 1, Johannesburg)

‘I’m being mentored at the moment, and it really helps to know the doctor is there.’ (Nurse 3, Johannesburg)

Participants’ willingness to contact their mentors when they experienced problems was also a result of trust and support that mentors built up when interacting with the DoH staff when they were asked for help:

‘And when my mentors come and evaluate me they don’t complain, they just guide.’ (Nurse 2, Limpopo)

‘They give us a listening ear … they do not come to find mistakes but to give us support and information to have team work spirit.’ (Deputy facility manager 2, Limpopo)

These quotes also point to the importance of developing personal relationships between mentors and mentees which in turn meant that participants felt comfortable enough to contact their mentors when they needed help.

A second important way in which the mentoring programme provided psychosocial support to the DoH staff was by facilitating an improvement in health workers’ self-efficacy for implementing their training and managing the initiation of ART without having doctors present:

‘I am like a doctor in the clinic as I can initiate.’ (Nurse 5, Limpopo)

‘I feel it empowered me through what I learnt and learn every day.’ (Nurse 7, Limpopo)

Finally, several nurses discussed how their mentors provided an emotional outlet, which allowed them to deal with the stress of taking on the responsibility of initiating and managing patients on ART:

‘And the mentors come and encourage us as sometimes we get depressed when patients stop taking drugs.’ (Nurse 2, Limpopo)

‘Their support is helpful, and if we are feeling stressed we offload.’ (Nurse 1, Limpopo)

## Discussion

The use of mentoring as an integral part of the implementing agency’s technical assistance programme appears to be successful in facilitating healthcare workers’ implementation of knowledge and skills gained through training. Based on our interpretation of evaluation interviews conducted with healthcare workers, we suggest several attributes of the programme contributed to this success.

Firstly, the mentoring programme used a multifaceted approach to supporting the health system. The implementing agency’s prior service delivery in the three provinces led them to identify key bottlenecks to ART implementation, and the mentoring programme was therefore designed to support the clinical, administrative and pharmacy facets of the health system simultaneously. Chien et al. studied a similar approach to mentoring in Malawi and found that supplementing clinical mentoring with support for facility and district-level systems improved the overall impact of the mentoring programme.^15^ Our findings suggest that health facility staff value these broader approaches to mentoring that include non-clinical staff as they may facilitate an overall improvement in services available to patients while simultaneously limiting constraints to the impact of clinical mentoring resulting from administrative or pharmacy-related problems. However, this approach does not address broader health system management issues, which Edwards et al. suggest may further improve patient outcomes.^6^

A second important attribute of the mentoring programme was the fact that the programme adopted a sequential proactive and reactive approach, which allowed mentors to leverage real-world problems faced by health workers to support their implementation of new knowledge and skills. The sequence of implementing the proactive and reactive aspects of the programme directly affected the effectiveness of mentoring. It was important that initial proactive mentoring occurred to build trust and relationships between mentors and mentees. Based on these relationships, health workers were comfortable enough to ask for help (i.e. to initiate reactive mentoring) when they needed it. Where mentors did not build relationships with mentees sufficiently, there appeared to be less willingness to ask for support. The development of trusting relationships between mentors and mentees as a prerequisite for successful mentoring relationships is a common finding in the mentoring literature.^3,16,17,18^ In the context of HSS in SSA, Edwards et al.^6^ found that mutual trust between implementing agency and Mozambique DoH staff based on an open and collaborative relationship was a critical enabling factor supporting the success of health management mentoring. The importance of developing trusting mentoring relationships during proactive mentoring phases of programme implementation should therefore be emphasised to mentors during the preparatory stages of these programmes.

Finally, mentors played an important role in providing psychosocial support to nurse mentees in particular. Psychosocial support was important because it helped nurses to develop the self-confidence to apply their NIMART training and to take responsibility for managing the ongoing care of patients on ART without direct supervision. There were several ways in which the mentoring programme was able to provide this type of support. Simply having the knowledge that mentors were available if they needed them enabled nurses to initiate patients onto ART without excessive anxiety about managing side effects or complications. Mentors also played an important role in providing psychosocial support by providing a means for nurses to deal with the stress of managing patients on ART. Here, the personal relationships between mentors and mentees were critical, and several participants noted how important their mentors’ caring and supportive attitudes were in helping them to gain confidence in implementing their new skills and knowledge. Although psychosocial support has long been recognised as a critical aspect of mentoring relationships, there is relatively little research conducted on the role of psychosocial support for mentees in health system-related interventions in LMICs.^8^ More broadly, however, Eller et al. found that a ‘caring personal relationship’ was an important aspect of effective mentoring relationships among nurses in the US, and the emotional support provided by mentors in other contexts has been found to improve mentees’ self-efficacy and emotional well-being.^18,19^ Our findings support the value of the mentor-mentee relationship as a source of psychosocial support. This suggests that the effectiveness of ART scale-up using mentoring programmes could potentially be improved by paying particular attention to the development of supportive relationships within these interventions.

There are some limitations to our study that may have affected our interpretation of the data. Firstly, our data only included interviews with the DoH staff; as such it is possible that we would have reached different conclusions if interview data were available from mentors and other implementing agency staff. Secondly, the authors are employed by the implementing agency, and while we made a conscious effort to remain objective, it is possible that our interpretations of the data were skewed by our personal investment in the mentoring programme.

In spite of these limitations, this study is among the first to examine the role of mentoring in strengthening HIV and TB programmes in South Africa. By identifying key factors supporting and limiting the success of the mentoring programme, this study may provide important lessons for the implementation of similar programmes elsewhere.

## Conclusion

Mentoring is a well-recognised and frequently used means of providing technical assistance in health-related interventions around the world.^6^ However, the likelihood of success of mentoring programmes depends on the dual role of mentors as supporting the proactive and reactive transfer of knowledge and skills to mentees, while simultaneously providing the psychosocial support necessary for mentees to develop the confidence to apply these in practice. Where this balance in mentors’ dual role is successfully achieved, mentoring is likely to be a powerful tool to improve healthcare globally.
